# Supplementary material for: Rational design of a microbial consortium of mucosal sugar utilizers reduces Clostridiodes difficile colonization
Source: Nat Commun. 2020 Oct 9;11:5104. doi: 10.1038/s41467-020-18928-1 (PMC7547075; doi:10.1038/s41467-020-18928-1)
Supplement: Supplementary file 1 — Supplementary Information [file 41467_2020_18928_MOESM1_ESM.pdf]

Supplementary Information

Rational design of a microbial consortium of mucosal sugar utilizers reduces *Clostridiodes difficile* colonization

Fátima C. Pereira, Kenneth Wasmund, Iva Cobankovic, Nico Jehmlich, Craig W. Herbold, Kang Soo Lee, Barbara Sziranyi, Cornelia Vesely, Thomas Decker, Roman Stocker, Benedikt Warth, Martin von Bergen, Michael Wagner and David Berry

Table of Contents:

Supplementary Figures 1-9………..…………………………………………………..2

Supplementary Tables 1-8………..…………………………………………..………14

Supplementary Figures

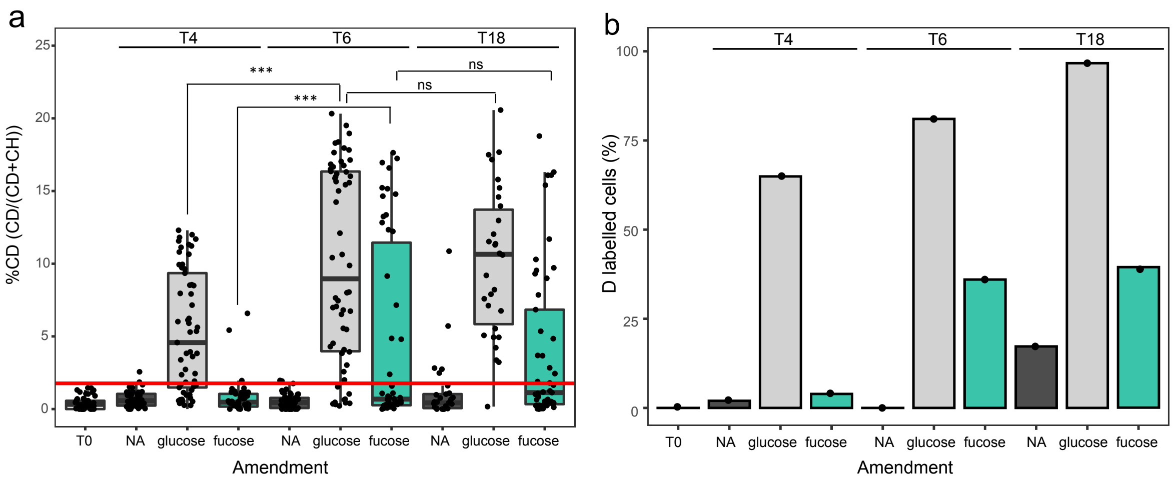

Supplementary Figure 1. Detection of D incorporation into microbiota cells at different time points of incubation with D_2O. A mouse colon community was incubated with glucose (positive control) or with fucose (as a representative of a mucosal sugar) in the presence of 50% D_2O for 0, 4, 6 and 18 hours. **a,** Deuterium incorporation (measured by %CD) of randomly-selected cells per microcosm at 0, 4, 6 and 18 hours post-amendment of either glucose or fucose. NA= no compound amended. The red horizontal line at 2.34 %CD indicates the threshold for considering a cell labeled (determined by calculating the mean + 3 SD of %CD in randomly selected cells from a sample incubated without addition of heavy water). Boxes represent median, first and third quartile. Whiskers extend to the highest and lowest values that are within one and a half times the interquartile range. ***p<0.0001; ns=non-significant; two-tailed Welch two-sample t test. **b,** Percentage of cells labeled (i.e., with %CD higher than threshold) per time point/condition as represented in **a**. After 4 hours of incubation we observed that a large percentage (64%) of cells had incorporated D to detectable levels in response to glucose, but not to fucose, which may be related with different pathways and energy gained from the catabolism of the two sugars. Levels of D incorporation (%CD) as well as the percentage of labeled cells increased by hour 6 in both microcosms. At a later time point (18 hours), levels of D incorporation as well as percentage of D labeled cells remained stable or increased only slightly, but we do observe an increase in the percentage of labeled cells in the negative control (no amendment), most likely due to cross-feeding. We therefore selected 6 hours as the incubation time for our experiments, as there was enough D labeling to be detected by Raman in response to both the positive control and mucosal sugar, while cross-feeding at this time point still appeared to be minimal. Source data are provided as a Source Data file.

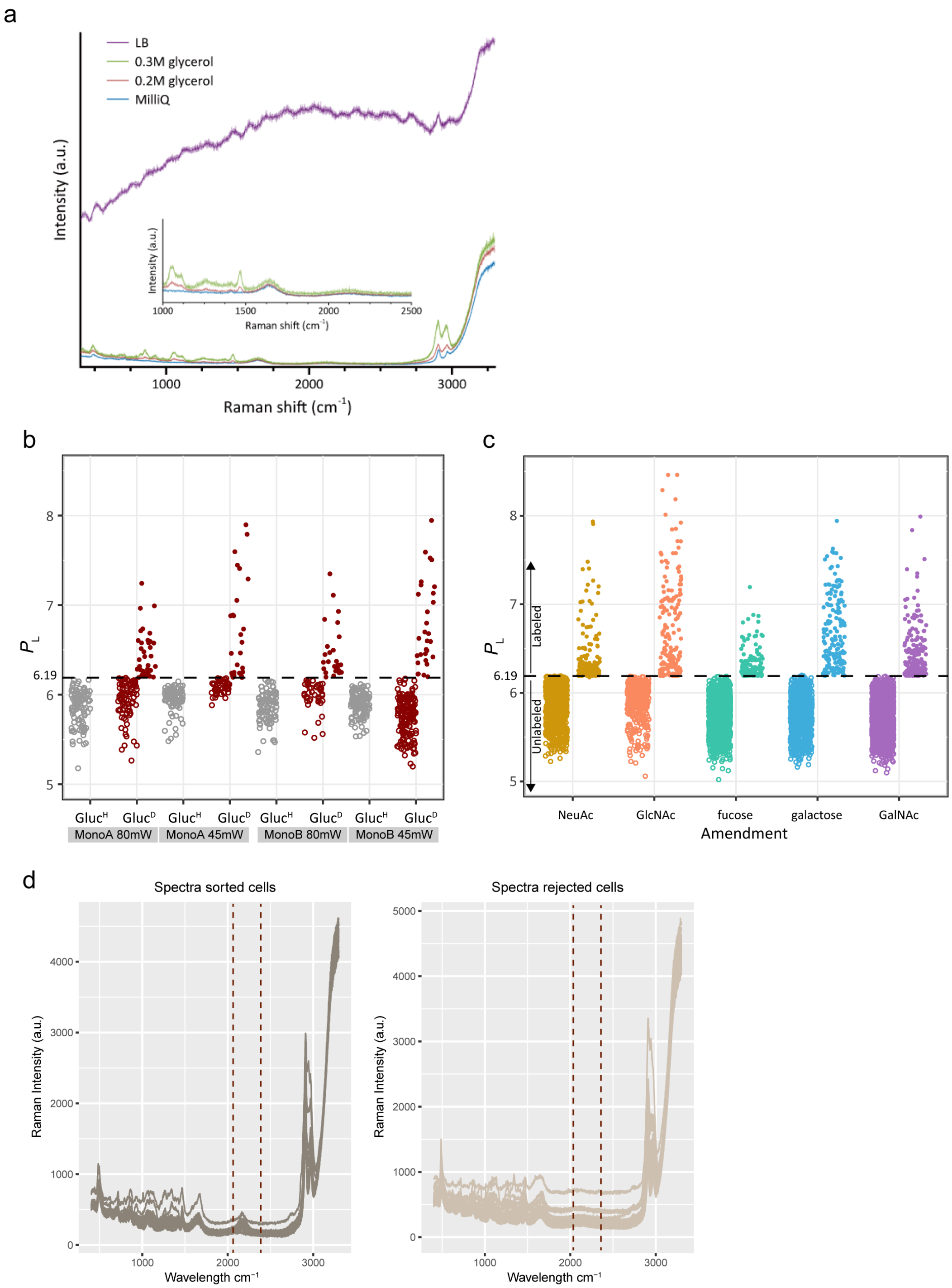

Supplementary Figure 2. RACS-analysis of microcosm samples. **a**, Background RACS spectra of MilliQ, 0.2 M Glycerol and 0.3 M glycerol (balanced with MilliQ) and LB medium. The addition of glycerol into MilliQ did not significantly change the intensity at the C–D peak (2,040–2,300 cm^{-1}), whereas the intensity at other spectral regions (<1,500 cm^{-1} and >2,700 cm^{-1}) increased. Inset shows the spectra of those three liquids at the spectral region 1,000–2,500 cm^{-1} for better discrimination. Some

liquids (e.g., LB) generate a highly fluorescent background that interferes with the identification of deuterated cells. Each spectrum represents the mean and SD of three independent measurements. **b,** P_L values of cells from glucose-supplemented microcosms of two different incubations (MonoA and MonoB), suspended in 0.3 M glycerol and analyzed by the two platform versions (version 1 and 2, see Methods) operating with two different laser intensities (45 mW and 80 mW). Control (Gluc^H; *n*= 446, grey) and test samples (Gluc^D; *n*=456) denote cells from colon microbiota incubated for 6 h in H_2O- or 50% D_2O-containing PBS, respectively. The dashed line denotes the threshold P_L value adopted based on these controls (P_L = 6.19). **c,** P_L values of cells from microcosms supplemented with different mucosal monosaccharides and mucin, suspended in 0.3 M glycerol. Cells with a $P_L > 6.19$ were considered labeled and sorted, while cells with a $P_L \leq 6.19$ were considered unlabeled and rejected. Dots represent all cells analyzed for each amendment from at least 6 independent sorts from samples originated from two independent experiments (MonoA and MonoB) (Supplementary Table 1). **d,** Exemplary Raman spectra of sorted (left panel) and rejected cells (right panel) from NeuAc-supplemented microcosms. The C-D peak-region of the Raman spectrum (between 2,040 and 2,300 cm^{-1}) is depicted using vertical dashed lines. Source data are provided as a Source Data file.

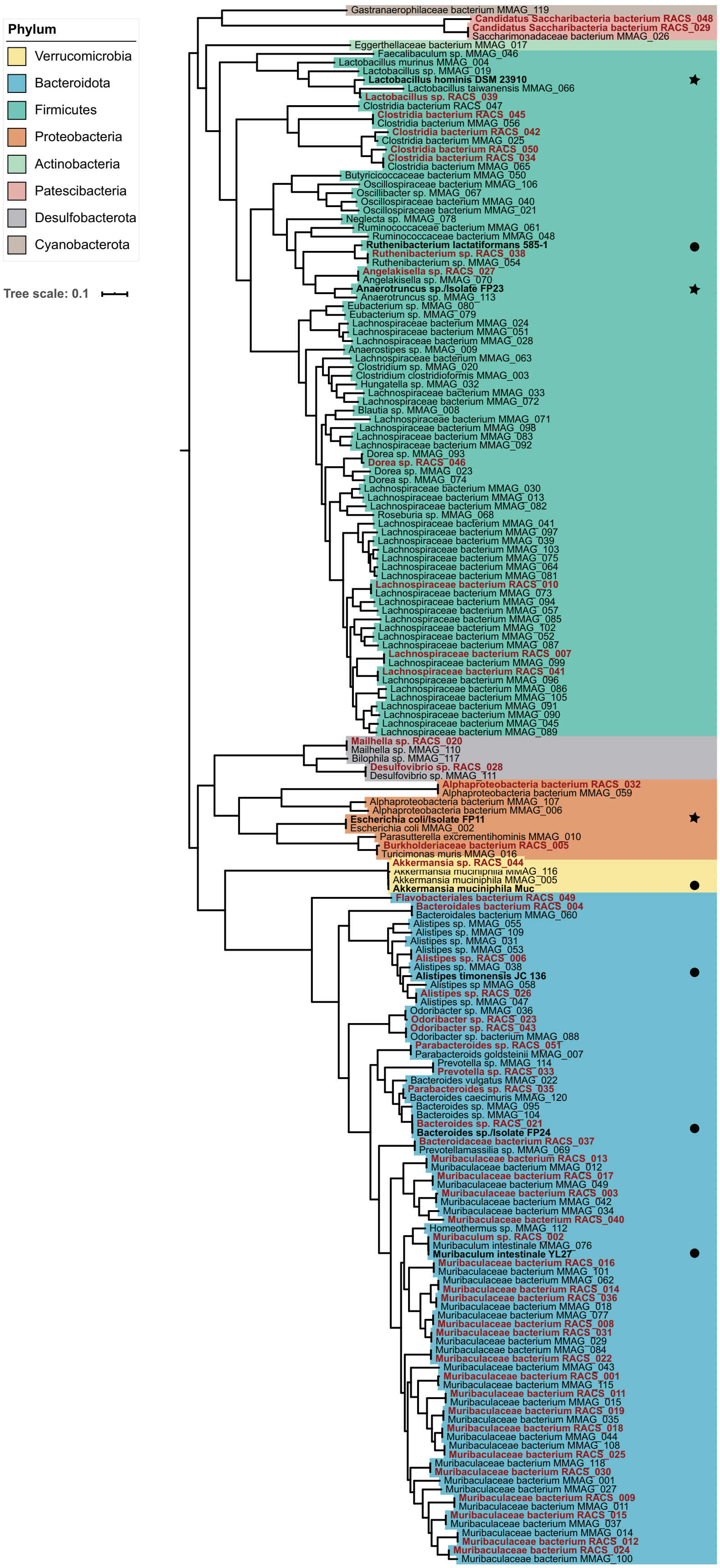
Phylum
Verrucomicrobia
Bacteroidota
Firmicutes
Proteobacteria
Actinobacteria
Patescibacteria
Desulfobacterota
Cyanobacterota
Tree scale: 0.1
Gastranaerophilaceae bacterium MMAG_119
Candidatus Saccharibacteria bacterium RACS_048
Candidatus Saccharibacteria bacterium RACS_029
Saccharimonadaceae bacterium MMAG_026
Eggerthellaceae bacterium MMAG_017
Faecalibaculum sp. MMAG_046
Lactobacillus murinus MMAG_004
Lactobacillus sp. MMAG_019
Lactobacillus hominis DSM 23910
Lactobacillus taiwanensis MMAG_066
Lactobacillus sp. RACS_039
Clostridia bacterium RACS_047
Clostridia bacterium RACS_045
Clostridia bacterium MMAG_056
Clostridia bacterium RACS_042
Clostridia bacterium MMAG_025
Clostridia bacterium RACS_050
Clostridia bacterium RACS_034
Clostridia bacterium MMAG_065
Butyricicoccaceae bacterium MMAG_050
Oscillospiraceae bacterium MMAG_106
Oscillibacter sp. MMAG_067
Oscillospiraceae bacterium MMAG_040
Oscillospiraceae bacterium MMAG_021
Neglecta sp. MMAG_078
Ruminococcaceae bacterium MMAG_061
Ruminococcaceae bacterium MMAG_048
Ruthenibacterium lactatiformans 585-1
Ruthenibacterium sp. RACS_038
Ruthenibacterium sp. MMAG_054
Angelakisella sp. RACS_027
Angelakisella sp. MMAG_070
Anaerotruncus sp./Isolate FP23
Anaerotruncus sp. MMAG_113
Eubacterium sp. MMAG_080
Eubacterium sp. MMAG_079
Lachnospiraceae bacterium MMAG_024
Lachnospiraceae bacterium MMAG_051
Lachnospiraceae bacterium MMAG_028
Anaerostipes sp. MMAG_009
Lachnospiraceae bacterium MMAG_063
Clostridium sp. MMAG_020
Clostridium clostridioformis MMAG_003
Hungatella sp. MMAG_032
Lachnospiraceae bacterium MMAG_033
Lachnospiraceae bacterium MMAG_072
Blautia sp. MMAG_008
Lachnospiraceae bacterium MMAG_071
Lachnospiraceae bacterium MMAG_098
Lachnospiraceae bacterium MMAG_083
Lachnospiraceae bacterium MMAG_092
Dorea sp. MMAG_093
Dorea sp. RACS_046
Dorea sp. MMAG_023
Dorea sp. MMAG_074
Lachnospiraceae bacterium MMAG_030
Lachnospiraceae bacterium MMAG_013
Lachnospiraceae bacterium MMAG_082
Roseburia sp. MMAG_068
Lachnospiraceae bacterium MMAG_041
Lachnospiraceae bacterium MMAG_097
Lachnospiraceae bacterium MMAG_039
Lachnospiraceae bacterium MMAG_103
Lachnospiraceae bacterium MMAG_075
Lachnospiraceae bacterium MMAG_064
Lachnospiraceae bacterium MMAG_081
Lachnospiraceae bacterium RACS_010
Lachnospiraceae bacterium MMAG_073
Lachnospiraceae bacterium MMAG_094
Lachnospiraceae bacterium MMAG_057
Lachnospiraceae bacterium MMAG_085
Lachnospiraceae bacterium MMAG_102
Lachnospiraceae bacterium MMAG_052
Lachnospiraceae bacterium MMAG_087
Lachnospiraceae bacterium RACS_007
Lachnospiraceae bacterium MMAG_099
Lachnospiraceae bacterium RACS_041
Lachnospiraceae bacterium MMAG_096
Lachnospiraceae bacterium MMAG_086
Lachnospiraceae bacterium MMAG_105
Lachnospiraceae bacterium MMAG_091
Lachnospiraceae bacterium MMAG_090
Lachnospiraceae bacterium MMAG_045
Lachnospiraceae bacterium MMAG_089
Mailhella sp. RACS_020
Mailhella sp. MMAG_110
Bilophila sp. MMAG_117
Desulfovibrio sp. RACS_028
Desulfovibrio sp. MMAG_111
Alphaproteobacteria bacterium RACS_032
Alphaproteobacteria bacterium MMAG_059
Alphaproteobacteria bacterium MMAG_107
Alphaproteobacteria bacterium MMAG_006
Escherichia coli/Isolate FP11
Escherichia coli MMAG_002
Parasutterella excrementihominis MMAG_010
Burkholderiaceae bacterium RACS_005
Turicimonas muris MMAG_016
Akkermansia sp. RACS_044
Akkermansia muciniphila MMAG_116
Akkermansia muciniphila MMAG_005
Akkermansia muciniphila Muc
Flavobacteriales bacterium RACS_049
Bacteroidales bacterium RACS_004
Bacteroidales bacterium MMAG_060
Alistipes sp. MMAG_055
Alistipes sp. MMAG_109
Alistipes sp. MMAG_031
Alistipes sp. MMAG_053
Alistipes sp. RACS_006
Alistipes sp. MMAG_038
Alistipes timonensis JC 136
Alistipes sp MMAG_058
Alistipes sp. RACS_026
Alistipes sp. MMAG_047
Odoribacter sp. MMAG_036
Odoribacter sp. RACS_023
Odoribacter sp. RACS_043
Odoribacter sp. bacterium MMAG_088
Parabacteroides sp. RACS_051
Parabacteroids goldsteinii MMAG_007
Prevotella sp. MMAG_114
Prevotella sp. RACS_033
Bacteroides vulgatus MMAG_022
Parabacteroides sp. RACS_035
Bacteroides caecimuris MMAG_120
Bacteroides sp. MMAG_095
Bacteroides sp. MMAG_104
Bacteroides sp. RACS_021
Bacteroides sp./Isolate FP24
Bacteroidaceae bacterium RACS_037
Prevotellamassilia sp. MMAG_069
Muribaculaceae bacterium RACS_013
Muribaculaceae bacterium MMAG_012
Muribaculaceae bacterium RACS_017
Muribaculaceae bacterium MMAG_049
Muribaculaceae bacterium RACS_003
Muribaculaceae bacterium MMAG_042
Muribaculaceae bacterium MMAG_034
Muribaculaceae bacterium RACS_040
Homeothermus sp. MMAG_112
Muribaculum sp. RACS_002
Muribaculum intestinale MMAG_076
Muribaculum intestinale YL27
Muribaculaceae bacterium RACS_016
Muribaculaceae bacterium MMAG_101
Muribaculaceae bacterium MMAG_062
Muribaculaceae bacterium RACS_014
Muribaculaceae bacterium RACS_036
Muribaculaceae bacterium MMAG_018
Muribaculaceae bacterium MMAG_077
Muribaculaceae bacterium RACS_008
Muribaculaceae bacterium RACS_031
Muribaculaceae bacterium MMAG_029
Muribaculaceae bacterium MMAG_084
Muribaculaceae bacterium RACS_022
Muribaculaceae bacterium MMAG_043
Muribaculaceae bacterium RACS_001
Muribaculaceae bacterium MMAG_115
Muribaculaceae bacterium RACS_011
Muribaculaceae bacterium MMAG_015
Muribaculaceae bacterium RACS_019
Muribaculaceae bacterium MMAG_035
Muribaculaceae bacterium RACS_018
Muribaculaceae bacterium MMAG_044
Muribaculaceae bacterium MMAG_108
Muribaculaceae bacterium RACS_025
Muribaculaceae bacterium MMAG_118
Muribaculaceae bacterium RACS_030
Muribaculaceae bacterium MMAG_001
Muribaculaceae bacterium MMAG_027
Muribaculaceae bacterium RACS_009
Muribaculaceae bacterium MMAG_011
Muribaculaceae bacterium RACS_015
Muribaculaceae bacterium MMAG_037
Muribaculaceae bacterium MMAG_014
Muribaculaceae bacterium RACS_012
Muribaculaceae bacterium RACS_024
Muribaculaceae bacterium MMAG_100

Supplementary Figure 3. Phylogenomic analysis of metagenome-assembled genomes recovered from sequencing of RACS sorted fractions (RACS_01 to RACS_051), from metagenomics sequencing of initial (T0), unsorted microcosms (MMAG001-MMAG120) and from the BacMix and BacMixC consortium members. The tree was generated with the maximum likelihood algorithm of FastTree using a concatenated alignment of 34 single copy marker gene protein sequences. The tree was rooted at mid-point and the scale bar represents 10% sequence divergence. Color shades denoted the bacterial phylum of each MAG. RACS-recovered MAGs are highlighted in red. BacMix and BacMixC genomes are highlighted in bold and marked with circles or stars, respectively.

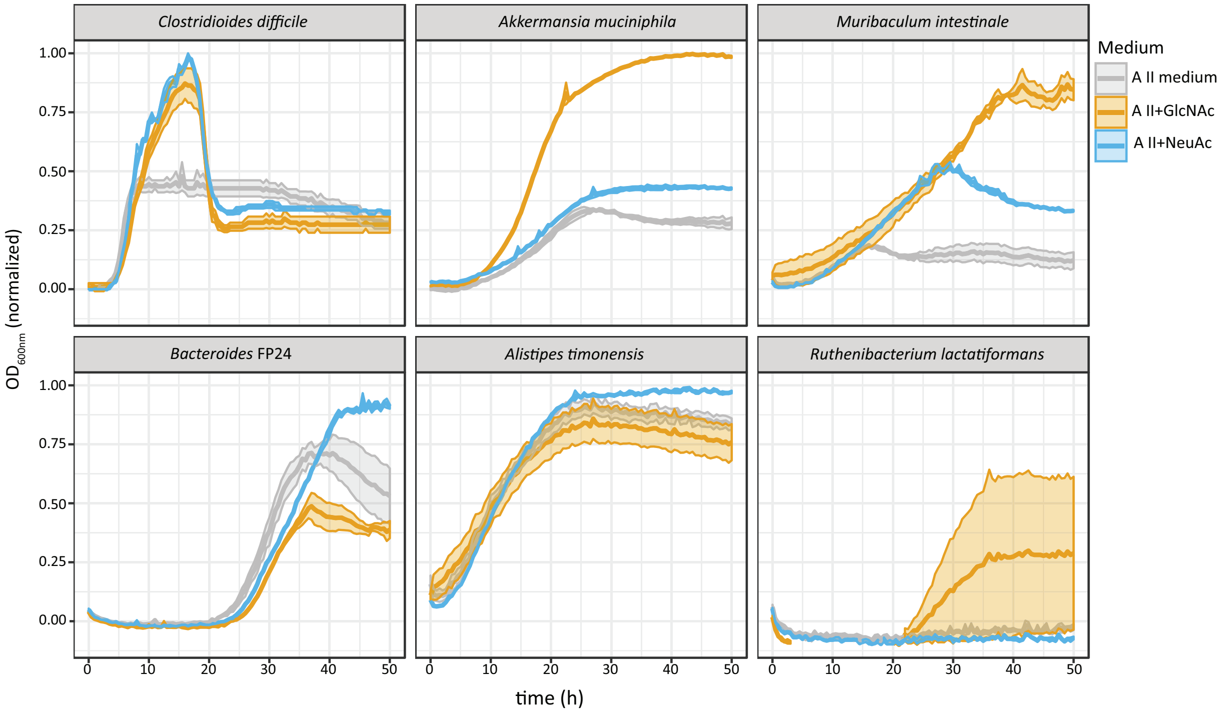

Supplementary Figure 4. Optical density (OD) measurements (600nm) of *C. difficile* and BacMix consortium members cultures grown independently in diluted A II medium non-supplemented or supplemented with the *O*-glycan sugars NeuAc (0.125% w/v) or GlcNAc (0.125% w/v). Line depicts mean values and shaded region s.e.m. from triplicates. Values were normalized to the maximum OD level attained by each individual strain. Source data are provided as a Source Data file.

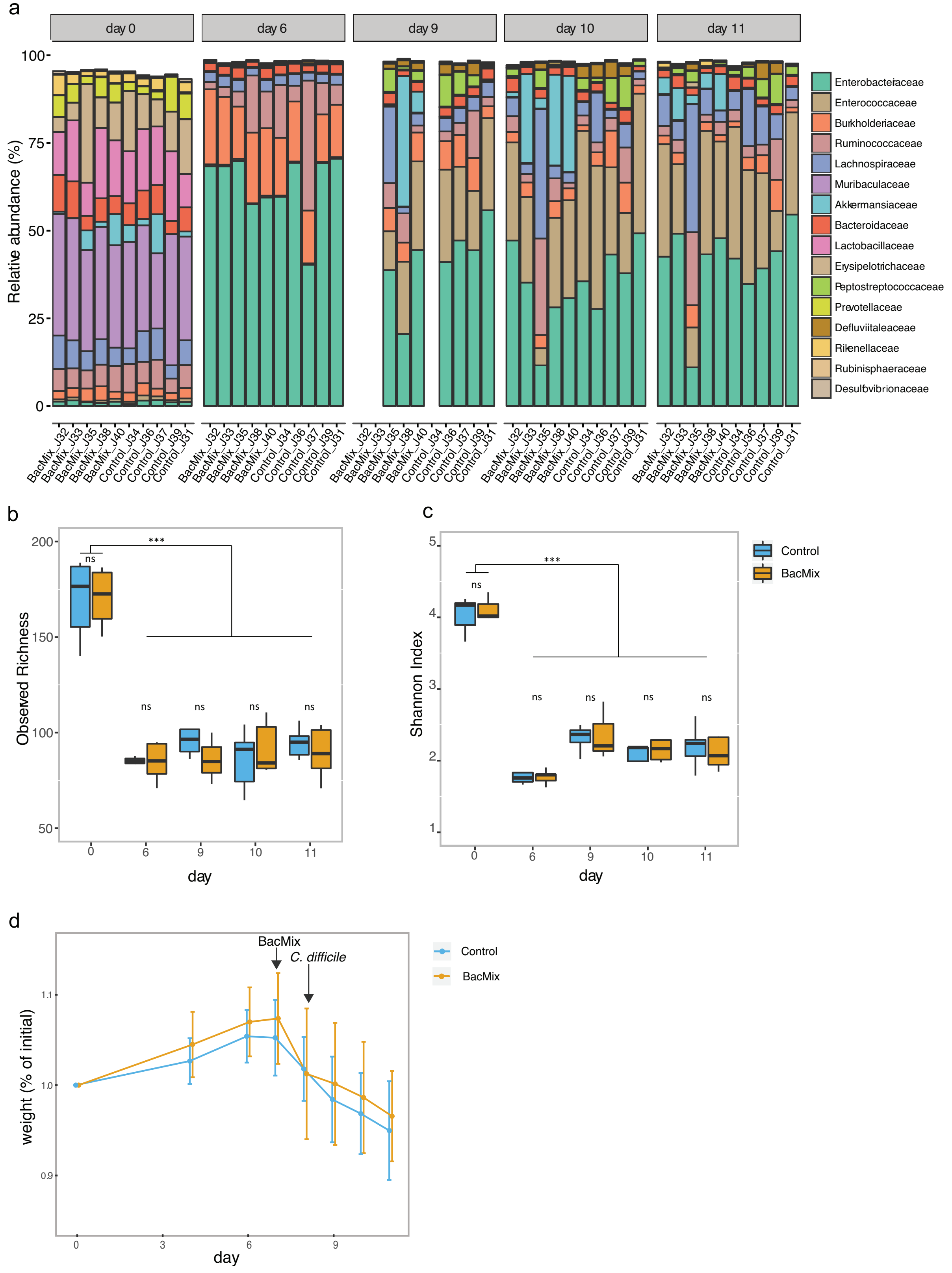

Supplementary Figure 5. a, Intestinal microbiota composition (at the family level) of

individual mice from Control and BacMix groups at the different indicated time points of the in vivo adoptive transfer experiment. Blank spaces indicate that no data is available because no fecal pellet from that mouse could be collected at that particular time point. **b, c,** Microbiota alpha-diversity metrics (Observed Richness (b) and Shannon index (c)) for the same time points and mice analyzed in **a**. Boxes represent median, first and third quartile and whiskers extend to the highest and lowest values that are within one and a half times the interquartile range (***p<0.0001 for both Observed Richness and Shannon Index; ns=non-significant; two-sided Mann-Whitney test). d, Evolution of mice weight throughout the in vivo adoptive transfer experiment (n=10/group). Source data are provided as a Source Data file.

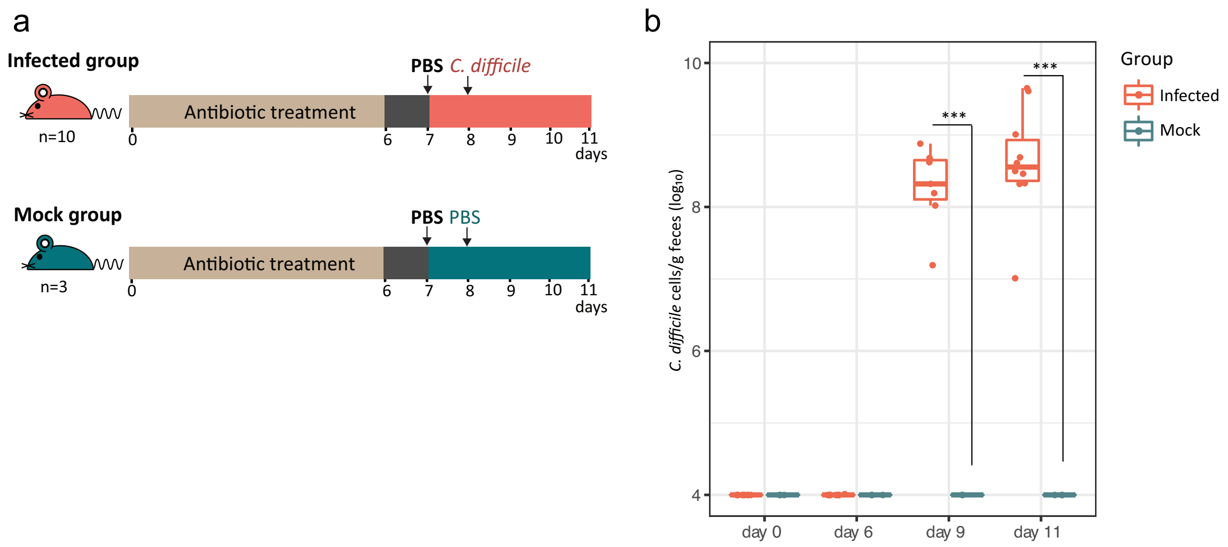

Supplementary Figure 6. *C. difficile* burden over time in infected and mock-infected mice. **a,** Outline of the infection experiment. **b,** *C. difficile* titers in fecal samples of antibiotic-treated mice infected with *C. difficile* or mock infected, assessed by qPCR. Boxes represent median, first and third quartile. Whiskers extend to the highest and lowest values that are within one and a half times the interquartile range; n=3 (mock group) and n=10 (infected group) (***p<0.0001; two-tailed Welch two sample t test). Detection limit: 10^4 cells/g feces. Source data are provided as a Source Data file.

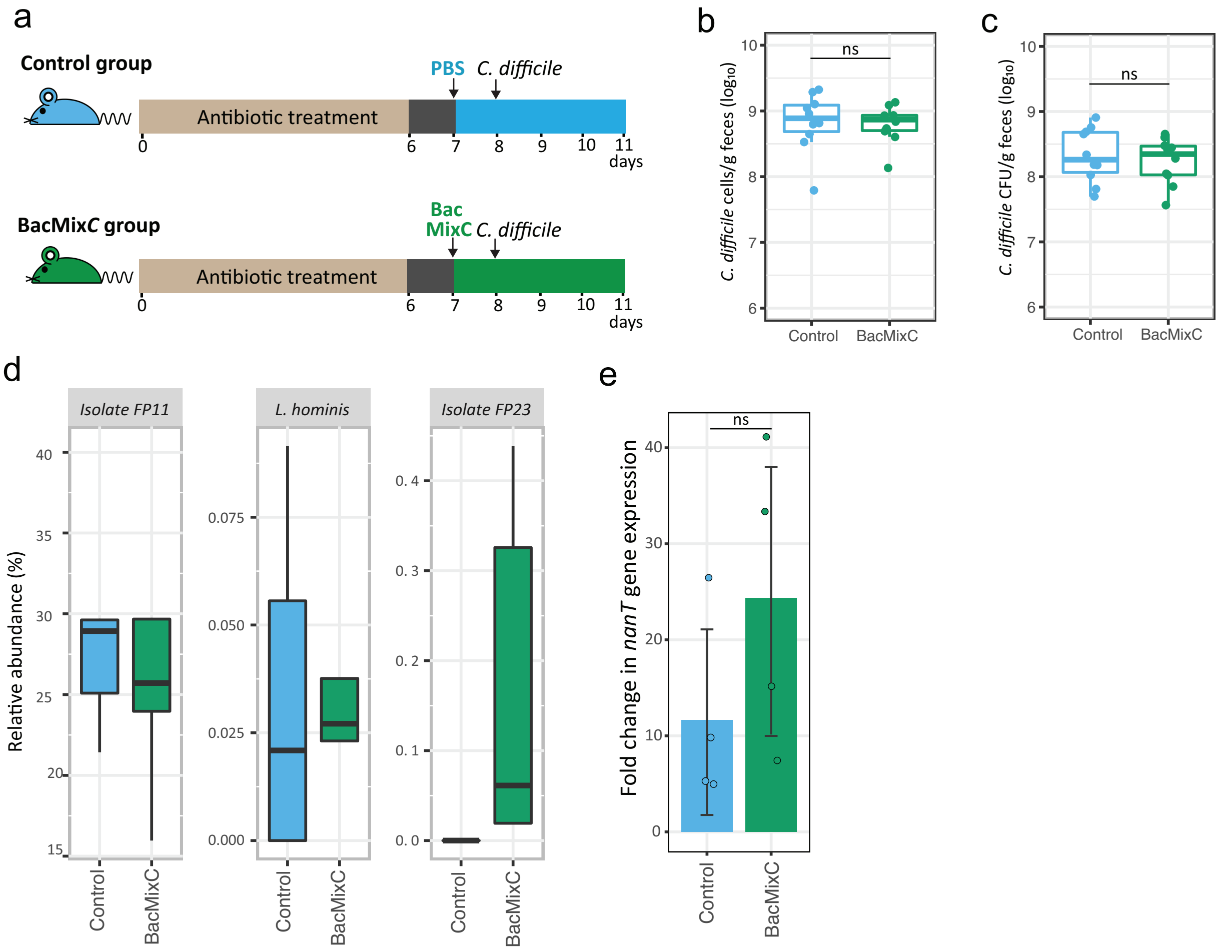

Supplementary Figure 7. Adoptive transfer of a control BacMixC consortium after antibiotic exposure of conventional mice does impact *C. difficile* levels. a, Schematic representation of the in vivo adoptive transfer experiment with the control bacteriothererapy BacMixC. **b**, **c,** Density of *C. difficile* three days post-infection in feces of antibiotic-treated control (blue) and BacMixC-recipient (green) mice determined by qPCR (**b**) or selective-medium plating (**c**) (*n*=10/group); ns: non-significant; two-sided Mann-Whitney test. **d**, Relative abundance of the three BacMixC members in fecal samples collected from control (blue) and BacMixC-recipient (green) mice (*n*=5/group) at day three post-infection as inferred from 16S rRNA gene amplicon sequencing. **e**, *C. difficile nanT* expression in fecal samples 2 days post-infection of antibiotic-treated control (blue) or and BacMixC-recipient (green) mice relative to growth in minimal medium containing 0.5% glucose (*n*=4/group); ns: non-significant, two-tailed Welch two-sample *t* test. In **b**, **c**, **d** and **e**, boxes represent median, first and third quartile, and whiskers extend to the highest and lowest values that are within one and a half times the interquartile range. Source data are provided as a Source Data file.

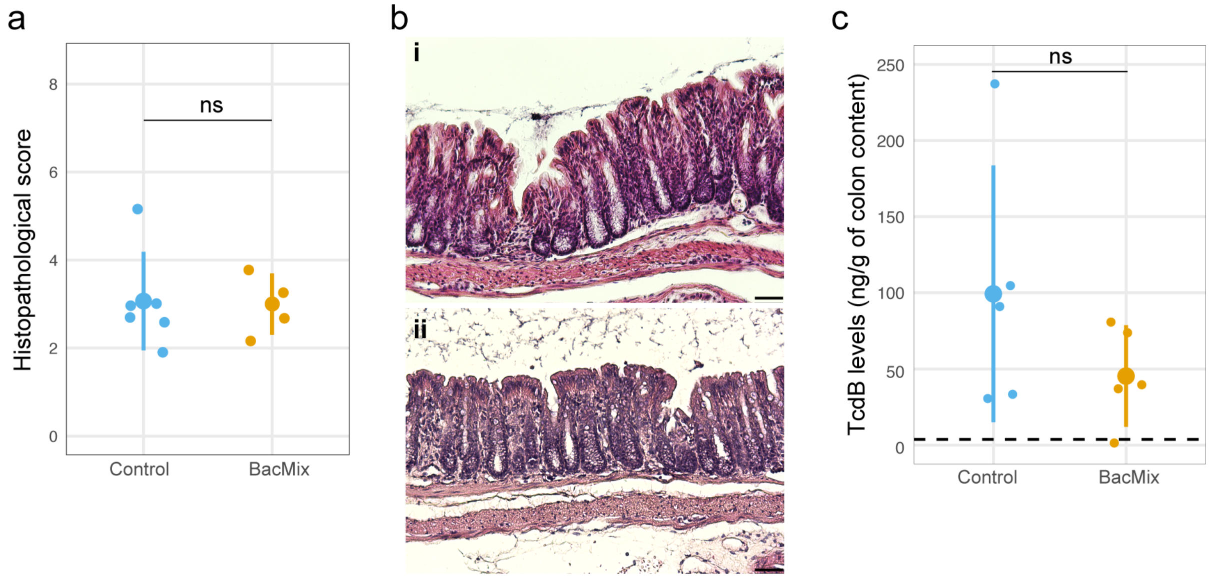

Supplementary Figure 8. Inflammation scores and *C. difficile* toxin levels in control and BacMix-recipient mice three days post-infection. a, Histopathology analysis was carried out on mouse colon tissue as described in the "Histology and histopathological scoring" section of Methods (*n*=6 for control group, *n*=4 for BacMix group). Small points represent the total histopathological score, large points represent the average score for each group and bars represent standard deviations; ns: non-significant, two-tailed Welch two-sample *t* test. **b**, Representative images of histologic sections stained with hematoxylin (blue/purple) and eosin (pink/red) from (i) control mice and (ii) BacMix recipient mice (*n*=5 per group). Scale bar: 100 μm **c**, Levels of TcdB were measured in colon contents of mice from the two different groups (*n*=5 per group). Small points represent TcdB levels for each mouse, large points represents the average level for each group and bars represent standard deviations; ns: non-significant, two-tailed Welch two-sample *t* test. Source data are provided as a Source Data file.

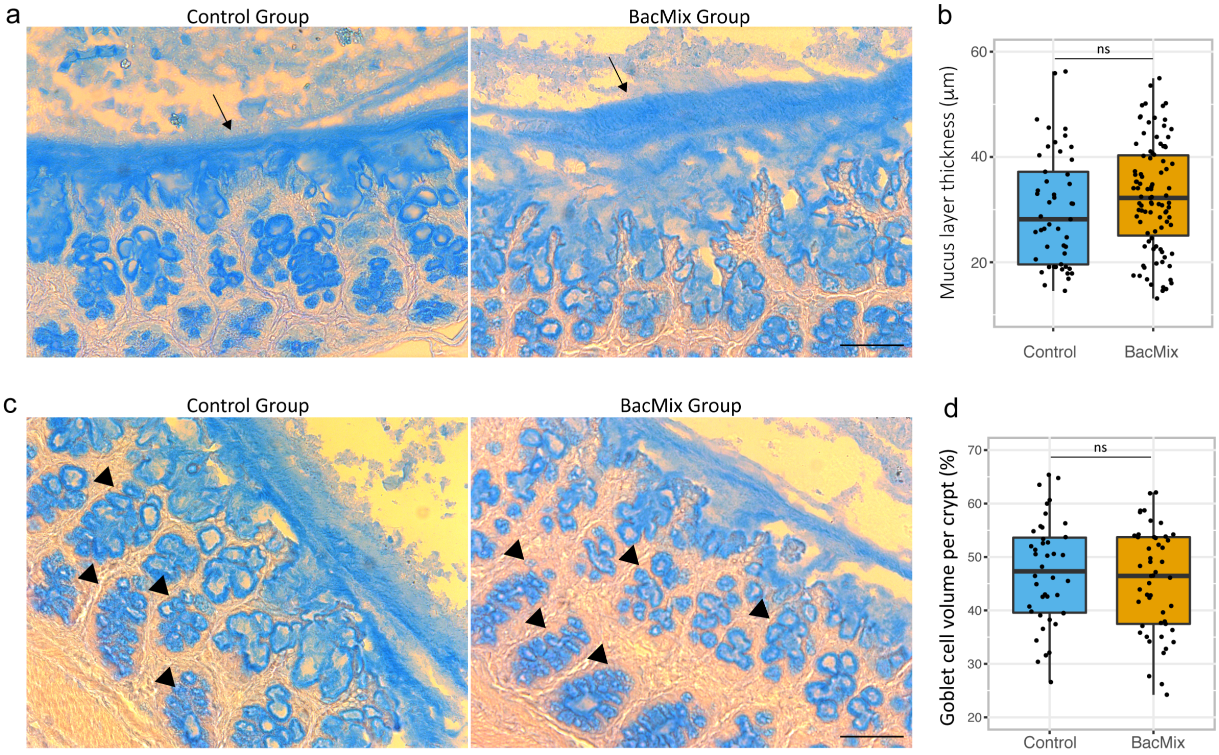

Supplementary Figure 9. Alcian Blue staining of paraffin embedded sections of the murine large intestine three days post-infection with *C. difficile*. **a,** Representative images of acquired fields. Stained mucus layer is indicated by arrows. **b,** Large intestine mucus layer thickness of control (blue) and BacMix-recipient (brown) mice. Measurements of a minimum of 50 independent areas obtained from different fields of view or within the same field of view at points 50μM apart were acquired per condition. Measurements and representative images were obtained from sections (*n*=7 for the control group; *n*=10 for the BacMix group) of the large intestine of 4 different animals per group. **c,** Representative images of fields of view used for goblet cell volume measurements. Stained goblet cells are indicated by arrowheads. **d,** Goblet cell volume per crypt, obtained by dividing the area covered by positive Alcian blue staining by the total area of the crypt. A total of 43-51 crypts per condition were analyzed from sections (*n*=8 for the control group; *n*=9 for the BacMix group) of the large intestine of 4 different animals per group. **b**, **d**: Boxes represent median, first and third quartile. Whiskers extend to the highest and lowest values that are within one and a half times the interquartile range; ns: non-significant, two-sided Mann-Whitney test. **a**, **c**: Scale bar= 50μM. Source data are provided as a Source Data file.

Supplementary Tables

Supplementary Table 1. Total cells analysed, D-labeled cells sorted and MAGs recovered using RACS.

Sorted fraction	Amendment	Incubation	Cells sorted	Cells rejected	% Cells sorted	# MAGs recovered*	% Cells sorted per amendment	# MAGs recovered per amendment
NanaB1	NeuAc	MonoB	28	146	19%	5	18%	41
NanaB2	NeuAc	MonoB	13	74	18%	2		
NanaB3	NeuAc	MonoB	14	61	23%	4		
Nana1	NeuAc	MonoA	23	133	17%	4		
Nana2	NeuAc	MonoA	15	147	10%	3		
Nana3	NeuAc	MonoA	22	105	21%	10		
NanaB5	NeuAc	MonoB	29	98	30%	13		
NanaB7	NeuAc	MonoB	6	67	9%	0		
GlcNAc4	GlcNAc	MonoA	20	88	23%	11	50%	31
GlcNAc5	GlcNAc	MonoA	38	74	51%	0		
GlcNAc6	GlcNAc	MonoA	32	57	56%	2		
GlcNAc12	GlcNAc	MonoB	34	61	56%	10		
GlcNAc13	GlcNAc	MonoB	33	56	59%	8		
GlcNAcB8	GlcNAc	MonoB	30	40	75%	0		
FucD1	Fucose	MonoA	14	258	5%	3	7%	23
FucD2	Fucose	MonoA	18	294	6%	9		
FucD3	Fucose	MonoA	19	293	6%	0		
FucA1	Fucose	MonoB	14	173	8%	0		
FucA2	Fucose	MonoB	33	337	10%	6		
FucA3	Fucose	MonoB	24	354	7%	5		
GalD1	Galactose	MonoA	25	217	12%	0	16%	36
GalD2	Galactose	MonoA	37	191	19%	4		
GalD3	Galactose	MonoA	16	137	12%	7		
GalA1	Galactose	MonoB	20	99	20%	5		
GalA2	Galactose	MonoB	16	96	17%	10		
GalA3	Galactose	MonoB	49	293	17%	10		
GalNAcD1	GalNAc	MonoA	50	308	16%	10	10%	43
GalNAcD2	GalNAc	MonoA	53	335	16%	9		
GalNAcD3	GalNAc	MonoA	14	283	5%	13		
GalNAcA1	GalNAc	MonoB	20	228	9%	0		
GalNAcA2	GalNAc	MonoB	23	264	9%	8		
GalNAcA3	GalNAc	MonoB	33	532	6%	3		
Neg_ctrl1	None	MonoA	0	103	-	0		
Neg_ctrl2	None	MonoB	0	106	-	0		

*completeness >50% & contamination<10%

Supplementary Table 2. Pairwise Average nucleotide identity[a] (ANI) between RACS-retrieved MAGs, metagenome-retrieved MAGs (MMAGs) and BacMix/BacMixC genomes.

query genome	reference genome	ANI[a,b]	alignment coverage[c]
**RACS-MAG / MMAG pairs**			
Muribaculaceae bacterium RACS_001	MMAG_115	0.999	0.956
Mulibaculum sp. RACS_002	MMAG_76	1.000	0.935
Muribaculaceae bacterium RACS_003	MMAG_42	0.999	1.001
Bacteroidales bacterium RACS_004	MMAG_59	1.000	0.994
Burkholderiaceae bacterium RACS_005	MMAG_16	0.999	0.933
Alistipes sp. RACS_006	MMAG_53	1.000	0.926
Lachnospiraceae bacterium RACS_007	MMAG_98	1.000	0.971
Muribaculaceae bacterium RACS_008	MMAG_77	0.997	0.950
Muribaculaceae bacterium RACS_009	MMAG_11	1.000	0.948
Lachnospiraceae bacterium RACS_010	MMAG_73	1.000	0.987
Muribaculaceae bacterium RACS_011	MMAG_15	1.000	0.896
Muribaculaceae bacterium RACS_012	MMAG_14	1.000	0.945
Muribaculaceae bacterium RACS_013	MMAG_12	0.999	0.894
Muribaculaceae bacterium RACS_014	MMAG_62	0.999	0.893
Muribaculaceae bacterium RACS_015	MMAG_37	0.997	0.944
Muribaculaceae bacterium RACS_016	MMAG_101	0.999	0.955
Muribaculaceae bacterium RACS_017	MMAG_49	1.000	0.953
Muribaculaceae bacterium RACS_018	MMAG_45	0.992	0.881
Muribaculaceae bacterium RACS_019	MMAG_35	1.000	0.920
Mailhela sp. RACS_020	MMAG_109	0.999	0.965
Bacteroides sp. RACS_021	MMAG_104	1.000	0.835
Muribaculaceae bacterium RACS_022	MMAG_84	1.000	0.883
Odoribacter sp. RACS_023	MMAG_36	1.000	0.942
Muribaculaceae bacterium RACS_024	MMAG_99	0.999	0.853
Muribaculaceae bacterium RACS_025	MMAG_107	0.999	0.969
Alistipes sp. RACS_026	MMAG_47	1.000	0.881
Angelakisella sp. RACS_027	MMAG_27	0.999	0.705
Desulfovibrio sp. RACS_028	MMAG_110	0.994	0.850
Candidatus Saccharibacteria bacterium RACS_029	MMAG_26	0.999	0.861
Muribaculaceae bacterium RACS_030	MMAG_118	1.000	0.839
Muribaculaceae bacterium RACS_031	MMAG_29	1.000	0.887
Alphaproteobacteria bacterium RACS_032	MMAG_61	1.000	0.959
Prevotella sp. RACS_033	MMAG_114	0.999	0.900
Clostridia bacterium RACS_034	MMAG_65	0.998	0.710
Muribaculaceae bacterium RACS_036	MMAG_18	1.000	0.936
Bacteroidaceae bacterium RACS_037	MMAG_72	0.999	0.934
Ruthenibacterium sp. RACS_038	MMAG_54	1.000	0.893
Lactobacillus sp. RACS_039	MMAG_66	0.999	0.387
Muribaculaceae bacterium RACS_040	MMAG_34	0.997	0.606
Lachnospiraceae bacterium RACS_041	MMAG_100	0.998	0.527
Clostridia bacterium RACS_042	MMAG_25	0.999	0.757
Odoribacter sp. RACS_043	MMAG_88	1.000	0.868
Clostridia bacterium RACS_045	MMAG_57	0.999	0.942
Dorea sp. RACS_046	MMAG_92	0.997	0.949
**BacMix member - Closest sorted organism**			
Akkermansia muciniphila strain Muc	Akkermansia sp. RACS_044	0.991	0.871
Muribaculum intestinale strain YL27	Muribaculum sp. RACS_002	0.999	0.951
Ruthenibacterium lactatiformans strain 585-1	Ruthenibacterium sp. RACS_038	0.841	0.140
Alistipes timonensis strain JC136	Alistipes sp RACS_026	0.849	0.300
Bacteroides sp. Isolate FP24	Bacteroides sp. RACS_021	0.998	0.946
**BacMixC member - Closest sorted organism[d]**			
Escherichia/Shigella sp. isolate FP11	-	-	-
Anaerotruncus colihominis Isolate FP23	-	-	-
Lactobacillus hominis strain DSM23910	-	-	-

[a] Richter, M., and Rossello-Mora, R. (2009) Shifting the genomic gold standard for the prokaryotic species definition. *Proc Natl Acad Sci U S A* **106**: 19126-19131

[b]Pairwise ANI was calculated for all genome pairs, but values are highly similar (less than 0.001 different) than from the ones shown here for one-way ANI.

[c] An alignment coverage of 0.1 was used as a cut-off

[d] no closest sorted organism could be found based on a cut-off alignment of 0.1

Supplementary Table 3. Number of hits for mucin-degrading enzymes (per sequenced Megabase pair) in scaffolds from each sorted fraction or microcosms metagenome.

		Number of hits per sequenced Mbp for each Mucin-degrading enzyme								
**Scaffold from Sorted fraction/Metagenome**	**Number of base pairs**	**Alpha-N-acetylglucosaminidase (EC 3.2.1.50)**	**Mucin-desulfating sulfatase (EC 3.1.6.14)**	**Sialidase (EC 3.2.1.18)**	**Alpha-L-fucosidase (EC 3.2.1.51), GH29 family**	**Alpha-L-fucosidase (EC 3.2.1.51), GH29 family, GH95**	**Beta-hexosaminidase (EC 3.2.1.52), GH3 family**	**Beta-hexosaminidase (EC 3.2.1.52), GH20 family**	**Alpha-galactosidase (EC 3.2.1.22), GH36 family**	**Beta-galactosidase (EC 3.2.1.23), GH2 family**
**Nana1**	7213673	0.69	0.83	0.28	1.66	0.55	2.08	1.25	0.42	2.77
**Nana2**	21548606	0.09	0.19	0.46	1.16	0.32	0.88	1.11	0.37	1.49
**Nana3**	121404695	0.07	0.08	0.17	0.86	0.17	1.26	0.49	0.63	2.14
**NanaB1**	35082564	0.29	0.11	0.31	1.23	0.31	0.83	0.94	0.37	1.94
**NanaB2**	23693278	0.30	0.17	0.51	1.94	0.30	1.10	1.18	0.59	2.53
**NanaB3**	26507725	0.34	0.15	0.45	1.36	0.34	0.94	1.09	0.72	2.11
**NanaB5**	222041970	0.08	0.06	0.13	0.81	0.15	0.94	0.43	0.68	1.82
**NanaB7**	24704093	0.08	0.04	0.36	1.46	0.36	0.85	0.69	0.73	2.55
**GlcNAc4**	115580502	0.13	0.04	0.14	0.80	0.18	1.12	0.37	0.67	1.79
**GlcNAc5**	14627058	0.00	0.21	0.07	0.62	0.00	0.34	0.41	0.68	1.57
**GlcNAc6**	22873773	0.13	0.09	0.35	0.74	0.17	0.92	0.57	0.70	2.36
**GlcNAc12**	109078488	0.16	0.17	0.28	1.16	0.28	1.31	0.83	0.51	2.52
**GlcNAc13**	87038963	0.26	0.16	0.21	1.38	0.33	1.37	1.10	0.44	3.00
**GlcNAcB8**	7967293	0.25	0.00	0.13	1.51	0.38	1.00	0.50	0.88	4.27
**FucA2**	59303066	0.08	0.07	0.27	1.11	0.19	1.18	0.61	0.51	2.28
**FucA3**	73391190	0.16	0.10	0.26	1.02	0.20	1.08	0.56	0.60	2.23
**FucD1**	22703868	0.18	0.13	0.13	1.45	0.22	1.37	0.66	0.57	1.98
**FucD2**	74145148	0.09	0.15	0.34	1.07	0.23	1.11	0.63	0.51	2.35
**FucD3**	11779936	0.08	0.25	0.42	1.02	0.17	0.93	1.19	0.59	2.80
**GalA1**	110060846	0.15	0.08	0.21	1.16	0.25	1.06	0.68	0.52	2.07
**GalA2**	55938931	0.11	0.11	0.27	0.97	0.29	1.25	0.66	0.32	1.68
**GalA3**	90463397	0.09	0.06	0.19	0.85	0.22	1.11	0.59	0.44	1.72
**GalD1**	11860294	0.08	0.59	0.51	1.18	0.17	0.84	1.35	0.25	2.11
**GalD2**	63170605	0.09	0.05	0.21	1.04	0.19	1.11	0.54	0.43	2.04
**GalD3**	78544204	0.14	0.06	0.20	0.92	0.19	1.08	0.46	0.57	1.99
**GalNAcA1**	10887300	0.09	0.09	0.18	2.30	0.37	1.19	1.01	0.46	3.95
**GalNAcA2**	75542222	0.09	0.13	0.30	1.09	0.26	1.10	0.57	0.49	1.05
**GalNAcA3**	21962081	0.09	0.14	0.09	1.14	0.23	1.23	0.41	0.50	1.91
**GalNAcD1**	106397377	0.15	0.08	0.23	1.18	0.23	1.11	0.77	0.55	1.00
**GalNAcD2**	55866911	0.14	0.13	0.30	1.07	0.27	1.16	0.72	0.38	4.22
**GalNAcD3**	90684184	0.08	0.08	0.22	0.86	0.23	1.04	0.55	0.47	2.60
**Metagenome_MonoA**	812432886	0.05	0.03	0.17	0.69	0.11	0.90	0.28	0.83	1.59
**Metagenome_MonoB**	694781510	0.06	0.04	0.19	0.77	0.14	1.11	0.29	0.97	1.72
	**Average sorted fractions:**	**0.15**	**0.15**	**0.26**	**1.16**	**0.25**	**1.09**	**0.74**	**0.53**	**2.29**
	**Average metagenome:**	**0.05**	**0.03**	**0.18**	**0.73**	**0.13**	**1.00**	**0.29**	**0.90**	**1.66**
	**Ratio Sorted Fraction/Metagenome:**	**2.8275**	**4.391**	**1.464**	**1.597**	**1.989**	**1.089**	**2.571**	**0.5953**	**1.3791**

Supplementary Table 4. Number of total and D-labeled peptides identified by Mass Spectrometry for each analyzed sample.

Microcosms	Amendment	total peptides	total matched peptides[a]	% total peptides matched	peptides with D incorporation	% peptides with D incorporated
NoAmendment_MonoA	NA	2336	2062	88%	4	0.17%
NoAmendment_MonoB	NA	4741	4406	93%	7	0.15%
NoAmendment_MonoC	NA	6111	5812	95%	6	0.10%
NeuAc_MonoA	NeuAc	8589	8204	96%	52	0.61%
NeuAc_MonoB	NeuAc	6808	6424	94%	31	0.46%
NeuAc_MonoC	NeuAc	16676	16354	98%	38	0.23%
GlcNAc_MonoA	GlcNAc	6456	6155	95%	36	0.56%
GlcNAc_MonoB	GlcNAc	9079	8624	95%	42	0.46%
GlcNAc_MonoC	GlcNAc	14975	14602	98%	62	0.41%
fucose_MonoA	Fucose	11428	11080	97%	45	0.39%
fucose_MonoB	Fucose	10080	9668	96%	34	0.34%
fucose_MonoC	Fucose	16456	16052	98%	56	0.34%
galactose_MonoA	Galactose	12425	12032	97%	98	0.79%
galactose_MonoB	Galactose	7464	7074	95%	93	1.25%
galactose_MonoC	Galactose	13555	13140	97%	52	0.38%
GalNAc_MonoA	GalNAc	9172	8865	97%	69	0.75%
GalNAc_MonoB	GalNAc	8326	7900	95%	54	0.65%
GalNAc_MonoC	GalNAc	14163	13799	97%	56	0.40%
mucin_MonoA	Mucin	9601	9226	96%	96	1.00%
mucin_MonoB	Mucin	8505	8064	95%	137	1.61%
mucin_MonoC	Mucin	11300	10845	96%	742	6.57%
			**Average match all samples**	**96%**		

Note: In all columns "peptides" refers to unique peptide sequences retrieved

[a] refers to the number of peptides that match a particular protein sequence from the microcosms metagenome database

Supplementary Table 5. Identity and relative isotope (D) abundance (RIA) of D labeled peptides that match selected proteins involved in mucosal sugar uptake.

Peptide Sequence	Protein Accessions[a]	RT[b]	Exp. m/z	Theo. m/z	Charge	RIA (D)
LPEHAIAK	N-ACETYLNEURAMINATE LYASE	22.18	439.7585	439.7584	2	6.4
LIGLDLGK	N-ACETYLNEURAMINATE LYASE	59.18	414.7631	414.7631	2	5.2
AFAEGDVETAR	N-ACETYLNEURAMINATE LYASE	33.26	583.2778	583.2778	2	4.9
MTEEIYDKAEFEK	L-FUCOSE ISOMERASE	46.38	816.8767	816.8767	2	3.1
ALAGEVAELIR	GLUCOSAMINE-6-PHOSPHATE DEAMINASE 1	72.11	571.3324	571.3324	2	11
MYETHHGMSK	GALACTOKINASE	14.9	407.5111	407.5111	3	6.3
EYGVTGSR	GALACTOKINASE	20.76	434.7118	434.7116	2	5.7
KYAFDDEGK	PUTATIVE ENDO-BETA-N-ACETYLGLUCOSAMINIDASE	23.54	536.7509	536.7509	2	4.9
FNLGDIVTSMIK	ALPHA-N-ACETYLGALACTOSAMINIDASE	104.6	669.3603	669.3603	2	4.6
LIGLDLGK	N-ACETYLNEURAMINATE LYASE	59.87	414.7631	414.7631	2	4.6
INESLDLGGVREPLPQLIESDLAIAQEAAR	N-ACETYLNEURAMINATE LYASE	104.7	1073.2403	1073.2403	3	4.1
AFLSPLEISLLPYGGR	N-ACETYLHEXOSAMINE 1-KINASE	103.47	866.9853	866.9853	2	4
LVLMDSVVK	GALACTOKINASE	59.68	502.2965	502.2965	2	3.5
M(Oxidation)LFGWVDENSAER	PUTATIVE ENDO-BETA-N-ACETYLGLUCOSAMINIDASE	64.87	785.3538	785.3538	2	3.5

[a] RAST Annotation of the protein(s) matched in metagenome database
[b] Retention time

Supplementary Table 6. Retention times and MS parameters of individual sugars.

Analytes	RTs	Parent ion		S-Lens	Product ions			
					Quantifier		Qualifier	
	[min]	[*m/z*]		[V]	[*m/z*]	CE [V]	[*m/z*]	CE [V]
**Galactose**	16	178.971	$[M-H]^-$	28	88	6	59	18
**GalNAc**	10.2	204.005	$[M+H-H_2O]^+$	55	138	13	186	6
**GlcNAc**	10.9	185.997	$[M+H-2H_2O]^+$	60	138	14	144	15
**Fucose**	5.8	163	$[M-H]^-$	50	89	5	102.9	5
**NeuAc**	25.8	308.480	$[M-H]^-$	60	86.9	18	170	16

Supplementary Table 7 – Oligonucleotides used in this study.

Gene	Forward primer [a]	Reverse primer [a]
**16S rRNA gene amplicon sequencing**		
16S rRNA	GCTATGCGCGAGCTGCCCTACGGGNGGCWGCAG	GCTATGCGCGAGCTGCGACTACHVGGGTATCTATCC
**qPCR primers targeting *C. difficile* genome**		
16S rRNA	GCAAGTTGAGCGATTTACTTCGGT	GTACTGGCTCACCTTTGATATTYAAGAG
dnaF	TCCATCTATTGCAGGGTGGT	CCCAACTCTTCGCTAAGCAC
nanA	GTGTAGATGGGGCAATTGGT	ATCCAGCCTCAACACCTTGT
nanT	ATCAATGGGACTTGCAACAGT	CAACTGAATTAAGCCCTGTCG
nagB	AAAGCTGTAAGTATGGGAATAGGT	GAACCAGGAACTTCTGGAGTTAT

[a]all sequences are in the 5´ to 3´ direction

Supplementary Table 8. GenBank accession numbers for the RACS MAGS.

Accession code	Description
WSLP00000000	Muribaculaceae bacterium RACS_001
WSLQ00000000	Muribaculum sp. RACS_002
WSLR00000000	Muribaculaceae bacterium RACS_003
WSLS00000000	Bacteroidales bacterium RACS_004
WSLT00000000	Burkholderiaceae bacterium RACS_005
WSLU00000000	Alistipes sp. RACS_006
WSLV00000000	Lachnospiraceae bacterium RACS_007
WSLW00000000	Muribaculaceae bacterium RACS_008
WSLX00000000	Muribaculaceae bacterium RACS_009
WSLY00000000	Lachnospiraceae bacterium RACS_010
WSLZ00000000	Muribaculaceae bacterium RACS_011
WSMA00000000	Muribaculaceae bacterium RACS_012
WSMB00000000	Muribaculaceae bacterium RACS_013
WSMC00000000	Muribaculaceae bacterium RACS_014
WSMD00000000	Muribaculaceae bacterium RACS_015
WSME00000000	Muribaculaceae bacterium RACS_016
WSMF00000000	Muribaculaceae bacterium RACS_017
WSMG00000000	Muribaculaceae bacterium RACS_018
WSMH00000000	Muribaculaceae bacterium RACS_019
WSMI00000000	Mailhella sp. RACS_020
WSMJ00000000	Bacteroides sp. RACS_021
WSMK00000000	Muribaculaceae bacterium RACS_022
WSML00000000	Odoribacter sp. RACS_023
WSMM00000000	Muribaculaceae bacterium RACS_024
WSMN00000000	Muribaculaceae bacterium RACS_025
WSMO00000000	Alistipes sp. RACS_026
WSMP00000000	Angelakisella sp. RACS_027
WSMQ00000000	Desulfovibrio sp. RACS_028
WSMR00000000	Candidatus Saccharibacteria bacterium RACS_029
WSMS00000000	Muribaculaceae bacterium RACS_030
WSMT00000000	Muribaculaceae bacterium RACS_031
WSMU00000000	Alphaproteobacteria bacterium RACS_032
WSMV00000000	Prevotella sp. RACS_033
WSMW00000000	Clostridia bacterium RACS_034
WSMX00000000	Parabacteroides sp. RACS_035
WSMY00000000	Muribaculaceae bacterium RACS_036
WSMZ00000000	Bacteroidaceae bacterium RACS_037
WSNA00000000	Ruthenibacterium sp. RACS_038
WSNB00000000	Lactobacillus sp. RACS_039
WSNC00000000	Muribaculaceae bacterium RACS_040
WSND00000000	Lachnospiraceae bacterium RACS_041
WSNE00000000	Clostridia bacterium RACS_042
WSNF00000000	Odoribacter sp. RACS_043
WSNG00000000	Akkermansia sp. RACS_044
WSNH00000000	Clostridia bacterium RACS_045
WSNI00000000	Dorea sp. RACS_046
WSNJ00000000	Clostridia bacterium RACS_047
WSNK00000000	Candidatus Saccharibacteria bacterium RACS_048
WSNL00000000	Flavobacteriales bacterium RACS_049
WSNM00000000	Clostridia bacterium RACS_050
WSNN00000000	Parabacteroides sp. RACS_051
