# Supplementary material for: Rational design of a microbial consortium of mucosal sugar utilizers reduces Clostridiodes difficile colonization
Source: Nat Commun. 2020 Oct 9;11:5104. doi: 10.1038/s41467-020-18928-1 (PMC7547075; doi:10.1038/s41467-020-18928-1)
Supplement: Supplementary file 3 — Description of Additional Supplementary Files [file 41467_2020_18928_MOESM3_ESM.docx]

**Description of Additional Supplementary Files**

**Rational design of a microbial consortium of mucosal sugar utilizers reduces *Clostridiodes difficile* colonization**

Fátima C. Pereira, Kenneth Wasmund, Iva Cobankovic, Nico Jehmlich, Craig W. Herbold, Kang Soo Lee, Barbara Sziranyi, Cornelia Vesely, Thomas Decker, Roman Stocker, Benedikt Warth, Martin von Bergen, Michael Wagner and David Berry

**Supplementary Data 1**

File name: Supplementary Data 1

Description: CheckM stats (completeness, contamination, strain heterogeneity, genome size) and taxonomic classification of MAGs obtained by direct shoutgun sequencing from initial microcosms.

**Supplementary Data 2**

File name: Supplementary Data 2

Description: Presence of genes involved in mucosal sugar utilization across the recovered MAGs and the BacMix consortium members.
