# Supplementary material for: Rational design of a microbial consortium of mucosal sugar utilizers reduces Clostridiodes difficile colonization
Source: Nat Commun. 2020 Oct 9;11:5104. doi: 10.1038/s41467-020-18928-1 (PMC7547075; doi:10.1038/s41467-020-18928-1)
Supplement: Supplementary file 6 — Reporting Summary [file 41467_2020_18928_MOESM6_ESM.pdf]

nature research

Corresponding author(s): Prof. David Berry

Last updated by author(s): Sep 14, 2020

Reporting Summary

Nature Research wishes to improve the reproducibility of the work that we publish. This form provides structure for consistency and transparency in reporting. For further information on Nature Research policies, see our Editorial Policies and the Editorial Policy Checklist.

Statistics

For all statistical analyses, confirm that the following items are present in the figure legend, table legend, main text, or Methods section.

n/a	Confirmed	
☐	☒	The exact sample size (*n*) for each experimental group/condition, given as a discrete number and unit of measurement
☐	☒	A statement on whether measurements were taken from distinct samples or whether the same sample was measured repeatedly
☐	☒	The statistical test(s) used AND whether they are one- or two-sided<br>*Only common tests should be described solely by name; describe more complex techniques in the Methods section.*
☐	☒	A description of all covariates tested
☐	☒	A description of any assumptions or corrections, such as tests of normality and adjustment for multiple comparisons
☐	☒	A full description of the statistical parameters including central tendency (e.g. means) or other basic estimates (e.g. regression coefficient) AND variation (e.g. standard deviation) or associated estimates of uncertainty (e.g. confidence intervals)
☐	☒	For null hypothesis testing, the test statistic (e.g. *F*, *t*, *r*) with confidence intervals, effect sizes, degrees of freedom and *P* value noted<br>*Give P values as exact values whenever suitable.*
☒	☐	For Bayesian analysis, information on the choice of priors and Markov chain Monte Carlo settings
☒	☐	For hierarchical and complex designs, identification of the appropriate level for tests and full reporting of outcomes
☒	☐	Estimates of effect sizes (e.g. Cohen's *d*, Pearson's *r*), indicating how they were calculated

Our web collection on statistics for biologists contains articles on many of the points above.

Software and code

Policy information about availability of computer code

Data collection	MATLAB GUI (graphical user interface) and code used in the RACS sorting (version 1; RACS_ver.1) is available at https://github.com/harubang2/MATLAB-platform-for-Raman-activated-cell-sorting-RACS.<br><br>MATLAB GUI (graphical user interface) and code used in the RACS sorting (version 2) is available at https://github.com/fatimapereira454/MATLAB-platform-for-Raman-activated-cell-sorting-RACS-version2.
Data analysis	We used Open source code and Software (available from previous publications):<br>-QIIME 1, UPARSE implemented in USEARCH v8.1.1861, RDPclassifier v2.12, Mothur v1.39.5, Silva database v132 and vegan package v2.4-3 (in R 3.4.0) were used for 16S rRNA gene sequence analyses.<br>-BBmap(v 34.00), SPAdes 3.11.1, MetaBAT 2 v2.12.1, CheckM 1.0.6, dRep 1.4.3, RAST 2.0, GTDB-Tk v0.1.3 and NCBIBlast 2.2.26 were used for genome and mini-metagenome sequencing and analyses.<br>-CheckM 1.0.6, FastTree 2.1.10, dRep 1.4.3 and iTOL v4 were used for phylogenomic analyses.<br>-MetaProSIP v2.3.2 (embedded in the Galaxy framework) was used for proteomic analyses.<br>-ImageJ 1.48v was used for image analysis.

For manuscripts utilizing custom algorithms or software that are central to the research but not yet described in published literature, software must be made available to editors and reviewers. We strongly encourage code deposition in a community repository (e.g. GitHub). See the Nature Research guidelines for submitting code & software for further information.

Data

Policy information about availability of data

All manuscripts must include a data availability statement. This statement should provide the following information, where applicable:

- Accession codes, unique identifiers, or web links for publicly available datasets
- A list of figures that have associated raw data
- A description of any restrictions on data availability

The data that supports the findings of this study are available from the corresponding author upon request. 16S rRNA gene sequence data have been deposited in the NCBI Sequence Read Archive (https://www.ncbi.nlm.nih.gov/sra) under SRP226368. Metagenomic data have been deposited in the NCBI Sequence Read Archive under SRP227836 and SRP144778 (for mucin amendment metagenomic data; Lee et al., 2019). RACS MAGs have been deposited as whole-genome shotgun projects at DDBJ/ENA/GenBank (https://www.ncbi.nlm.nih.gov/genbank/) under the accessions WSLP00000000- WSNN00000000 (Supplementary Table 8).

The mass spectrometry proteomics data have been deposited to the ProteomeXchange Consortium (http://proteomecentral.proteomexchange.org) via the PRIDE (Perez-Riverol et al., 2019) partner repository with the dataset identifier PXD015215. Source data are provided with this paper.

All of the custom codes used in this study can be accessed upon reasonable request from the corresponding author. MATLAB GUI (graphical user interface) and code used in the RACS sorting (version 1; RACS_ver.1) is available at https://github.com/harubang2/MATLAB-platform-for-Raman-activated-cell-sorting-RACS. MATLAB GUI (graphical user interface) and code used in the RACS sorting (version 2) is available at https://github.com/fatimapereira454/MATLAB-platform-for-Raman-activated-cell-sorting-RACS-version2.

Field-specific reporting

Please select the one below that is the best fit for your research. If you are not sure, read the appropriate sections before making your selection.

☒ Life sciences ☐ Behavioural & social sciences ☐ Ecological, evolutionary & environmental sciences

For a reference copy of the document with all sections, see nature.com/documents/nr-reporting-summary-flat.pdf

Life sciences study design

All studies must disclose on these points even when the disclosure is negative.

Sample size	For microcosms experiments, colon contents from 3 animals were used per incubation. For RACS sorting the sample size was constrained by the number of collected cells after the sorting. We used all samples collected during the sorting in our developed platform.<br>For C. difficile animal experiments, no sample size calculation was performed. We used 5 animals per condition, with the exception of a mock infected control for which we used 3 animals. The animal numbers were chosen based on feasibility. The number of animals chosen was adequate, as it allowed us to reproducibly detect analogous differences among treatments/groups.
Data exclusions	No data was excluded from the analyses.
Replication	Microcosms experiments were established in three independent experiments (Incubations MonoA, MonoB and MonoC), and replication of results was successful.<br>C. difficile animal experiments were performed in two independent experiments. Replication of results was successful.
Randomization	Animals were randomly allocated to treatment groups. For Mass Spectrometry analyses, both sample preparation and sample measurements were randomized.
Blinding	Our sorting platform works in a fully automated manner, we were totally blinded to group allocation during data collection and/or analysis. Mass Spectrometry data collection was performed blindly. Bioinformatic analysis was not performed blindly.<br>For experiments involving animals, animals were randomly allocated to treatment groups. Blinding during the experiment was not possible, as different groups of animals had to receive a different treatment. Administration of treatment and routine handling of animals as well as sample collection were performed by the same researcher, to minimize stress induced to animals. Histopathological analysis of mouse colon sections was carried out by an independent investigator that performed the scoring in a blinded manner.

Reporting for specific materials, systems and methods

We require information from authors about some types of materials, experimental systems and methods used in many studies. Here, indicate whether each material, system or method listed is relevant to your study. If you are not sure if a list item applies to your research, read the appropriate section before selecting a response.

Materials & experimental systems

n/a	Involved in the study
☒	☐ Antibodies
☒	☐ Eukaryotic cell lines
☒	☐ Palaeontology and archaeology
☐	☒ Animals and other organisms
☒	☐ Human research participants
☒	☐ Clinical data
☒	☐ Dual use research of concern

Methods

n/a	Involved in the study
☒	☐ ChIP-seq
☒	☐ Flow cytometry
☒	☐ MRI-based neuroimaging

Animals and other organisms

Policy information about studies involving animals; ARRIVE guidelines recommended for reporting animal research

Laboratory animals

For microcosm experiments, a total of 9 C57BL/6N mice (4 males, 5 females) bred at the Max F. Perutz Laboratories, University of Vienna, were used (3 mice per independent experiment). Each experiment included either 1 male and 2 females (Incubations MonoA and MonoB), or 1 female and 2 males (Incubations MonoC)
For C. difficile animal experiments, 33 female C57BL/6N mice, 6-8 weeks old were purchase from Janvier Labs.

Animals were kept in isolated, ventilated cages under specific pathogen-free conditions at the animal facility of the Max F. Perutz Laboratories, University of Vienna, Austria, with controlled temperature of 21 ± 1°C and humidity of 50 ± 10%, in a 12-h light/dark cycle. Mice received a standard diet (V1124-300; Ssniff, Soest, Germany) and autoclaved water ad libitum. For C. difficile animal experiments, at the time of BacMix and BacMixC administration, the mouse diet was switched from a standard diet to a isocaloric polysaccharide-deficient chow (Ssniff, Soest, Germany; Riva et al., 2019) with sucrose but no cellulose or starch.

Wild animals

The study did not involve wild animals.

Field-collected samples

The study did not involve field-collected samples.

Ethics oversight

All animal experiments were performed at the Max F. Perutz Laboratories of the University of Vienna, Austria. All experiments were discussed and approved by the University of Veterinary Medicine, Vienna, Austria, and conducted in accordance with protocols approved by the Federal Ministry for Education, Science and Research of the Republic of Austria under the license number BMWF-66.006/0001-WF/V/3b/2016.

Note that full information on the approval of the study protocol must also be provided in the manuscript.
